# Patient characteristics and incidence of in-hospital events in spontaneous coronary artery dissection and suspected concurrent Takotsubo syndrome: A report of the iSCAD registry

**DOI:** 10.1016/j.ahjo.2026.100793

**Published:** 2026-05-09

**Authors:** Gerald Chi, Esther S.H. Kim, Heather L. Gornik, Anna Grodzinsky, Bryan J. Wells, Jennifer Lewey, Daniella Kadian-Dodov, Angela M. Taylor, Connie N. Hess, Lori M. Tam, Rina Mauricio, Stanislav Henkin, Kathryn J. Lindley, James L. Orford, Sonia Tolani, Fahad Alkhalfan, Cassandra Reyes, C. Michael Gibson, Katherine K. Leon, Sahar Naderi, Nandita S. Scott, Malissa J. Wood

**Affiliations:** a PERFUSE Study Group, Division of Cardiovascular Medicine, Beth Israel Deaconess Medical Center, Harvard Medical School, Boston, MA, USA; b Division of Cardiovascular Medicine, Vanderbilt University Medical Center, Nashville, TN, USA; c Currently Sanger Heart and Vascular Institute, Atrium Health, Charlotte, NC, USA; d Division of Cardiovascular Medicine and Harrington Heart & Vascular Institute, University Hospitals, Cleveland, OH, USA; e Department of Cardiovascular Diseases, Saint Luke's Mid America Heart Institute, Kansas City, MO, USA; f Department of Medicine, Division of Cardiology, Emory University School of Medicine, Atlanta, GA, USA; g Division of Cardiology, University of Pennsylvania Perelman School of Medicine, Philadelphia, PA, USA; h The Zena and Michael A. Wiener Cardiovascular Institute, Icahn School of Medicine at Mount Sinai, New York, NY, USA; i Division of Cardiovascular Medicine, University of Virginia Medical Center, Charlottesville, VA, USA; j Division of Cardiology, Department of Medicine, University of Colorado School of Medicine, Aurora, CO, USA; k Providence Heart Institute, Portland, OR, USA; l Division of Cardiology, University of Texas Southwestern Medical Center, Dallas, TX, USA; m Heart and Vascular Center, Dartmouth-Hitchcock Medical Center, Lebanon, NH, USA; n Currently Gonda Vascular Center, Mayo Clinic, Rochester, MN, USA; o Cardiovascular Division, Washington University in St Louis, St Louis, MO, USA; p Currently Division of Cardiovascular Medicine, Vanderbilt University Medical Center, Nashville, TN, USA; q Intermountain Medical Center Heart Institute, Salt Lake City, UT, USA; r Division of Cardiology, Columbia University Irving Medical Center/New York-Presbyterian Hospital, New York, NY, USA; s SCAD Alliance, Alexandria, VA, USA; t Department of Cardiology, Kaiser Permanente San Francisco Medical Center, San Francisco, CA, USA; u Division of Cardiology, Massachusetts General Hospital, Harvard Medical School, Boston, MA, USA; v Florida State University College of Medicine, Tallahassee, FL, USA

**Keywords:** Spontaneous coronary artery dissection, Takotsubo cardiomyopathy, Myocardial infarction, Cardiac arrhythmias, Prognosis

## Abstract

**Background and objectives:**

Takotsubo syndrome (TTS) and spontaneous coronary artery dissection (SCAD) are both more common in middle-aged women and often share similar triggers, such as emotional or physical stress. This study aimed to examine the characteristics and in-hospital prognosis of patients with SCAD and possible concomitant TTS.

**Methods:**

Patients from the iSCAD Registry with completed questionnaires, case report forms, and available coronary angiography and ventriculography adjudicated by a core lab were included. In the absence of follow-up imaging confirming resolution of the wall motion abnormalities a definitive diagnosis of TTS cannot be established, therefore this pattern will be referred to throughout the manuscript as TTS-like angiographic pattern (TVP). Coexistent TVP was defined by classic apical ballooning or wall motion abnormalities discordant with the dissected coronary territory. In-hospital events included recurrent myocardial infarction (MI), cerebrovascular accident, heart failure (HF) requiring diuretics, and new arrhythmias. Patient characteristics and in-hospital events were compared between TVP and non-TVP groups.

**Results:**

Among 259 study participants, 44 (17.0%) were identified with coexistent TVP. There were no significant differences in age, cardiovascular risk factors, anxiety or depression history, recreational substance use, emotional or physical stressors, or extracoronary vascular abnormalities between the TVP and non-TVP groups. Patients with TVP were more likely to present with ST-segment elevation MI, left ventricular dysfunction, left anterior descending artery involvement, and abnormal myocardial perfusion compared to non-TVP patients. Furthermore, TVP patients had a higher incidence of experiencing in-hospital events (34.1% vs. 13.1%; *p* < 0.001), primarily driven by new arrhythmias (21.4% vs. 6.4%; *p* = 0.002) and HF (10.0% vs. 2.0%; *p* = 0.010). After multivariable adjustment, coexistent TVP remained associated with in-hospital events.

**Conclusions:**

In this SCAD cohort, patients with concurrent TVP had a greater risk of new arrhythmias or HF during hospitalization.

**Study registration:**

URL: http://www.clinicaltrials.gov. Identifier: NCT04496687.

## 1 Introduction

Spontaneous coronary artery dissection (SCAD) represents an important cause of myocardial infarction (MI) with non-obstructive coronary arteries resulting from a non-traumatic, non-iatrogenic separation of the coronary arterial wall [1]. Takotsubo syndrome (TVP), an acute cardiac condition mimicking MI, is characterized by transient reversible ventricular wall motion abnormalities (WMAs) that usually extend beyond a single epicardial coronary distribution [2]. While the precise pathophysiology underlying these distinct entities is unclear, SCAD and TTS share several features, including a predilection for women, emotional or physical stress as the predisposing stressor, acute chest pain as a frequent clinical presentation, ischemic electrocardiographic changes, positive cardiac biomarkers, and spontaneous recovery as the natural history observed in certain patients. Further, it has been posited that SCAD may develop as a consequence of arterial injury due to increased torsion and wall stress at the hinge point between ventricular segments with various degrees of WMA noted in TTS [3], [4], [5]. Conversely, SCAD may potentially serve as the precipitating trigger for the development of TTS [6]. Previous studies have reported missed diagnosis of SCAD among patients with a provisional diagnosis of TTS [7]. Although the association of TTS with SCAD has been described, the prognostic implications of coexistent TTS in patients with SCAD remain unexplored. The objective of this study is to investigate the characteristics and incidence of in-hospital events among patients with SCAD and suspected concomitant TTS.

## 2 Methods

The International Spontaneous Coronary Artery Dissection (iSCAD) Registry (ClinicalTrials.gov Identifier: NCT04496687) is a multicenter data repository aiming to advance the understanding of SCAD and improve patient outcomes. Patients meeting the following criteria were enrolled: (1) age 18 or older, and (2) SCAD suspected, diagnosed, or with a documented history based on coronary angiography. Patients were ineligible if any of the following applied: 1) coronary dissection in association with atherosclerosis or as a result of iatrogenic injury; 2) inability to provide informed consent; 3) inability to complete study-related patient questionnaires; and 4) inability to understand and complete patient questionnaires independently. SCAD-related medical history, presentation, echocardiography, management, and in-hospital events were recorded in case report forms and completed by site investigators. Sociodemographic characteristics, other general medical history, family history, information related to circumstances preceding SCAD events, health behaviors, and psychosocial factors were patient-reported and collected from online questionnaires. Coronary angiography and left ventriculography were independently adjudicated by the core laboratory, including assessment of SCAD lesion characteristics, Thrombolysis in Myocardial Infarction (TIMI) Flow Grade of the dissected epicardial coronary artery, TIMI Myocardial Perfusion Grade of the dissected epicardial coronary territory, and regional WMA on ventriculography [8]. All data were coordinated by the PERFUSE Study Group. Each enrolling site and the PERFUSE Study Group center received Institutional Review Board approval to conduct this research.

The present study included iSCAD Registry participants with completed patient questionnaires, case report forms, and available coronary angiography and left ventriculography. TVP was defined as a mismatch between the perfusion territory of the dissected coronary artery and the left ventricular (LV) segment affected by WMA through visual assessment. TVP was classified into classic TVP and TVP variants based on the distribution of WMA. Classic TVP was defined as the presence of WMA presenting as apical ballooning. TVP variants were defined as the presence of non-apical WMA discordant to dissected coronary territory with apical sparing and included midventricular, basal, and focal types. Echocardiography was performed at time of hospitalization for SCAD, however in the absence of an echo core lab echocardiograms were not utilized for diagnosis of takotsubo. Echocardiographic data including left ventricular ejection fraction and presence of LV dysfunction (defined as LVEF <50%) was abstracted by site investigators. In the absence of follow-up imaging confirming resolution of the wall motion abnormalities a definitive diagnosis of TVP cannot be established, therefore this pattern will be referred to throughout the manuscript as TVP-like angiographic pattern (TVP). Extracoronary vascular abnormality was defined as the presence of multifocal or focal fibromuscular dysplasia, or arterial dissection, aneurysm, or pseudoaneurysm. In-hospital events were defined as the composite of the occurrence of recurrent MI, cerebrovascular accident (CVA), heart failure (HF) requiring diuretics, or new arrhythmias during hospitalization for the index SCAD. New arrhythmias were classified into ventricular fibrillation, ventricular tachycardia, and other types of arrhythmias.

### 2.1 Statistical analysis

Continuous data were expressed as means and standard deviations, and categorical data were expressed as frequency counts and percentages. Patient characteristics, presentations, angiography findings, and in-hospital events were compared between patients with SCAD with and without coexistent TVP using the chi-squared test for categorical variables and Kruskal-Wallis test for continuous variables, respectively. Multivariable logistic regression was performed to explore factors associated with the occurrence of in-hospital events, including patient characteristics, clinical presentations, LV dysfunction, angiographic findings, and coexistent TVP. A *p*-value threshold of <0.25 was used to select covariates for entry into the final model. Collinearity among TVP, LAD involvement, STEMI presentation, and LV dysfunction were was assessed using the variance inflation factor (VIF). A threshold of VIF >5 was used to indicate potential collinearity, and VIF >10 was considered evidence of severe collinearity warranting variable removal. The prognostic value of TVP for in-hospital events was further evaluated within the stratum of patients who had SCAD involvement in the LAD. As TVP variants could represent stunned myocardium reperfused by healing dissected arteries, a sensitivity analysis was performed to compare patients with classic TVP against all other patients (i.e., TVP variants and non-TVP). Missing data were excluded from the analyses. All analyses were performed using two-sided tests at a significance level of 0.05 with the SAS version 9.4 (SAS Institute, Inc., Cary, North Carolina, United States).

## 3 Results

Among 659 study participants who had completed patient questionnaires, case report forms, and available coronary angiography, 259 (39.3%) underwent left ventriculography. Among these, TVP was identified in 44 (17.0%) patients (classic, midventricular, and focal pattern: 88.6%, 2.3%, and 9.1%, respectively). Patient characteristics, clinical presentations, imaging findings, and management were summarized in Table 1.

**Table 1:** Patient characteristics, clinical presentations, imaging findings, and management for SCAD in TVP group versus non-TVP group.

Variable	Total (***N*** = 259)	TVP (***N*** = 44)	Non-TVP (***N*** = 215)	P-value
Patient characteristics				
Age at first SCAD, Mean (SD)	50.2 (10.5)	52.2 (11.4)	49.8 (10.3)	0.265
Body mass index, Mean (SD)	28.0 (8.9)	27.6 (6.9)	28.1 (9.3)	0.710
Female	245/258 (95.0%)	43/43 (100.0%)	202 (94.0%)	0.098
White	237 (91.5%)	41 (93.2%)	196 (91.2%)	0.662
Smoking	68/257 (26.5%)	15 (34.1%)	53/213 (24.9%)	0.207
Diabetes	3 (1.2%)	0 (0.0%)	3 (1.4%)	0.431
Hyperlipidemia	38 (14.7%)	7 (15.9%)	31 (14.4%)	0.799
Hypertension	67 (25.9%)	15 (34.1%)	52 (24.2%)	0.172
Previous myocardial infarction	42/236 (17.8%)	5/40 (12.5%)	37/196 (18.9%)	0.337
Anxiety	76 (29.3%)	15 (34.1%)	61 (28.4%)	0.448
Depression	56 (21.6%)	10 (22.7%)	46 (21.4%)	0.845
Recreational substance use	28/257 (10.9%)	4 (9.1%)	24/213 (11.3%)	0.673
Emotional stress when experiencing SCAD	33 (12.7%)	5 (11.4%)	28 (13.0%)	0.764
Physical stress when experiencing SCAD	31 (12.0%)	6 (13.6%)	25 (11.6%)	0.708
Extracoronary vascular abnormalities	124/227 (54.6%)	23/39 (59.0%)	101/188 (53.7%)	0.549
Clinical presentations				
ST-segment elevation MI	86 (33.2%)	23 (52.3%)	63 (29.3%)	0.003
Non-ST-segment elevation MI	129 (49.8%)	14 (31.8%)	115 (53.5%)	0.009
Unstable angina	46 (17.8%)	5 (11.4%)	41 (19.1%)	0.223
Cardiogenic shock	2 (0.8%)	0 (0.0%)	2 (0.9%)	0.521
Cardiac arrest	19 (7.3%)	6 (13.6%)	13 (6.0%)	0.079
Echocardiographic findings				
LVEF, Mean (SD)	53.8 (10.8)	45.6 (10.4)	55.6 (10.0)	<0.001
LV dysfunction (LVEF <50%)	57/207 (27.5%)	25/38 (65.8%)	32/169 (18.9%)	<0.001
Angiographic findings				
Involvement of left main artery	1 (0.4%)	1 (2.3%)	0 (0.0%)	0.027
Involvement of left anterior descending artery	160 (61.8%)	39 (88.6%)	121 (56.3%)	<0.001
Involvement of left circumflex artery	90 (34.7%)	7 (15.9%)	83 (38.6%)	0.004
Involvement of right coronary artery	64 (24.7%)	5 (11.4%)	59 (27.4%)	0.024
Multivessel involvement	49 (18.9%)	5 (11.4%)	44 (20.5%)	0.160
Type 1 SCAD	29 (11.2%)	3 (6.8%)	26 (12.1%)	0.312
Type 2 SCAD	190 (73.4%)	32 (72.7%)	158 (73.5%)	0.917
Type 3 SCAD	63 (24.3%)	11 (25.0%)	52 (24.2%)	0.909
Abnormal TIMI Flow Grade	120 (46.3%)	23 (52.3%)	97 (45.1%)	0.386
Abnormal TIMI Myocardial Perfusion Grade	137 (52.9%)	30 (68.2%)	107 (49.8%)	0.026
LVG findings				
WMA in anterobasal segment	27/256 (10.5%)	6 (13.6%)	21/212 (9.9%)	0.463
WMA in anterolateral segment	98/256 (38.3%)	37 (84.1%)	61/212 (28.8%)	<0.001
WMA in apical segment	121/255 (47.5%)	41 (93.2%)	80/211 (37.9%)	<0.001
WMA in diaphragmatic segment	91/256 (35.5%)	34 (77.3%)	57/212 (26.9%)	<0.001
WMA in posterobasal segment	24/256 (9.4%)	2 (4.5%)	22/212 (10.4%)	0.227
Management				
Management for SCAD				0.005
Medical therapy only	186/254 (73.2%)	24/43 (55.8%)	162/211 (76.8%)	
Revascularization	68/254 (26.8%)	19/43 (44.2%)	49/211 (23.2%)	
Medications at discharge				
ACEI/ARB	79 (30.5%)	22 (50.0%)	57 (26.5%)	0.002
Beta blocker	215 (83.0%)	36 (81.8%)	179 (83.3%)	0.817
Calcium channel blocker	27 (10.4%)	3 (6.8%)	24 (11.2%)	0.390
Diuretic	18 (6.9%)	5 (11.4%)	13 (6.0%)	0.206

There were no significant differences in age, body mass index, sex, race, cardiovascular risk factors, history of anxiety or depression, recreational substance use, emotional or physical stressors, or extracoronary vascular abnormalities between the TVP and non-TVP groups. In comparison to the non-TVP groups, those with TVP demonstrated higher prevalence of ST-segment elevation MI (52.3% vs. 29.3%; *p* = 0.003) and left ventricular dysfunction (65.8% vs. 18.9%; *p* < 0.001). Involvement of the left anterior descending artery (LAD) (88.6% vs. 56.3%; p < 0.001) and impairment of microvascular perfusion, as measured by TIMI Myocardial Perfusion Grade (68.2% vs. 49.8%; *p* = 0.026), were also more common in TVP patients. The two groups exhibited similar percentages of multivessel involvement, SCAD angiographic types, and abnormal coronary flow in the dissected artery. WMAs in anterolateral, apical, and diaphragmatic segments were more frequently observed in TVP patients. Revascularization was more frequently performed in TVP patients than in non-TVP patients (44.2% vs. 23.2%; *p* = 0.005). At discharge, TVP patients were more likely to receive angiotensin-converting enzyme inhibitors (ACEIs) or angiotensin receptor blockers (ARBs) (50.0% vs. 26.5%; *p* = 0.002), while the rates of beta-blocker, calcium channel blocker, and diuretic usage were comparable between the two groups.

**Fig. 1:**
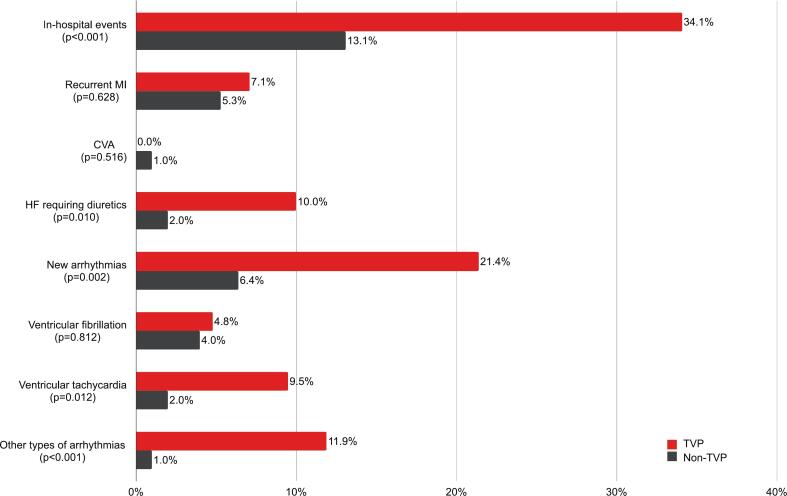
Incidence of in-hospital events among patients with SCAD, comparing those with classic TVP versus non-TVP. Abbreviations: MI = myocardial infarction; CVA = cerebrovascular accident; HF = heart failure; TVP = Takotsubo syndrome-like ventriculographic pattern; SCAD = spontaneous coronary artery dissection.

As presented in Fig. 1, TVP patients had a greater risk of experiencing in-hospital events compared to non-TVP patients (34.1% vs. 13.1%; *p* < 0.001). This elevated risk was primarily attributed to the occurrence of new arrhythmias (21.4% vs. 6.4%; p = 0.002) and HF requiring treatment with diuretics (10.0% vs. 2.0%; *p* = 0.010). Specifically, a larger proportion of TVP patients experienced ventricular tachycardia (9.5% vs. 2.0%; *p* = 0.012) and other types of arrhythmias, such as asymptomatic sinus pauses, bigeminy, premature atrial contractions, premature ventricular contractions, and atrial fibrillation requiring cardioversion (11.9% vs. 1.0%; *p* < 0.001), compared to the non-TVP group. There was no significant difference in recurrent MI or CVA between the two groups. After multivariable adjustment, coexistent TVP (odds ratio [OR], 5.74; 95% confidence interval [CI], 2.10 to 15.70; p < 0.001) and cardiac arrest (OR, 6.75; 95% CI, 1.94 to 23.57; *p* = 0.003) were associated with the occurrence of in-hospital events. There was no substantial collinearity among TVP, LAD involvement, STEMI presentation, and LV dysfunction (all VIF <5). Within the stratum of patients who had SCAD involvement in the LAD, TVP remained independently associated with in-hospital events (OR, 9.30; 95% CI, 2.44 to 35.49; *p* = 0.001).

**Fig. 2:**
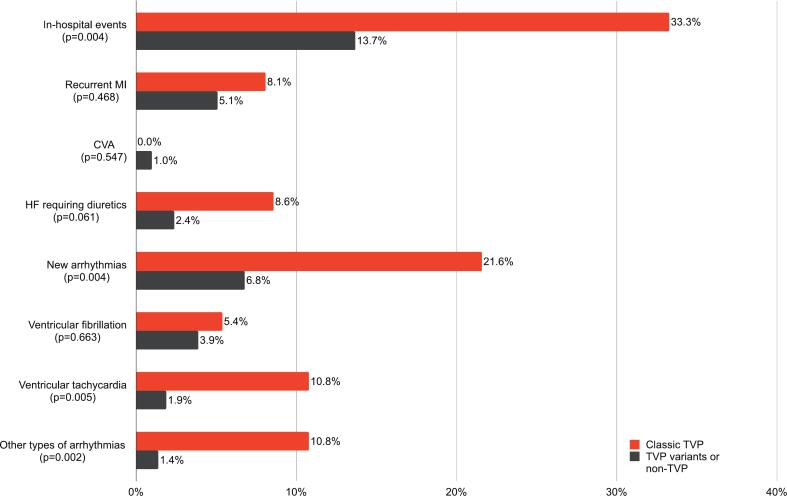
Incidence of in-hospital events among patients with SCAD, comparing those with classic TVP versus TVP variants or non-TVP. Abbreviations: MI = myocardial infarction; CVA = cerebrovascular accident; HF = heart failure; TVP = Takotsubo syndrome-like ventriculographic pattern; SCAD = spontaneous coronary artery dissection.

The sensitivity analysis, which compared patients with classic TVP to those with TVP variants or non-TVP patients, yielded consistent results with the main findings concerning patient characteristics, clinical presentations, imaging findings, and management (Table 2). Classic TVP was associated with a higher risk of in-hospital events compared to TVP variants or non-TVP patients (33.3% vs. 13.7%; *p* < 0.004), mainly driven by the occurrence of new arrhythmias (21.6% vs. 6.8%; *p* = 0.004) (Fig. 2). After multivariable adjustment, coexistent classic TVP (OR, 4.79; 95% CI, 1.69 to 13.59; *p* = 0.003) and cardiac arrest (OR, 7.22; 95% CI, 2.12 to 24.57; *p* = 0.002) were associated with the occurrence of in-hospital events.

## 4 Discussion

The study demonstrated that a considerable portion of patients with SCAD displayed WMAs on left ventriculography consistent with TVP, aligning with previous research from another registry and nationwide administrative claims-based database [5], [9]. The coexistence of SCAD and TVP has been hypothesized through various mechanisms. Both conditions may be associated with underlying physical or emotional stressors, suggesting a common pathophysiological pathway involving an adrenergic-mediated response characterized by a stress-induced surge in catecholamines. Alternatively, TVP may develop as a consequence of SCAD, possibly due to transient widespread ischemia or emotional distress. Conversely, TVP itself may contribute to the development of SCAD, as the contrasting WMAs observed in TVP could induce a hinge-point distortion within the coronary arteries, ultimately leading to dissection [3], [4], [5]. However, rather than implying mechanistic independence from the underlying ischemic substrate, TVP may represent a high-risk ventriculographic phenotype within the clinical spectrum of SCAD. Despite sharing similar patient characteristics with the registry population as a whole, the subset with TVP exhibited remarkable distinctions including a higher propensity for presentation with ST-segment elevation MI, LV dysfunction, LAD involvement, and abnormal myocardial perfusion in the dissected territory. Moreover, this subset was characterized by a heightened risk of in-hospital events, primarily attributed to the development of new arrhythmias and HF. These findings underscore the importance of recognizing this subgroup of SCAD patients with concurrent TVP manifestations, as they may require additional monitoring and management.

Several studies have compared the patient characteristics and clinical outcomes between TVP and SCAD by pooling data from separate registries. In a study comparing patients enrolled from the International Takotsubo Registry to patients enrolled from the Canadian SCAD Cohort Study, TVP patients exhibited a higher prevalence of cardiovascular risk factors, physical triggers, ST-segment elevation MI, and LV dysfunction, along with a higher rate of in-hospital mortality [10]. Similar results were observed in another study that compared TVP patients enrolled from a single-center registry to SCAD patients enrolled from a nationwide Spanish registry, revealing that TVP was associated with a less favorable in-hospital prognosis in terms of death or HF [11]. The present study extends the literature by examining the characteristics and prognostic significance of coexistent TVP among an angiographically confirmed SCAD cohort. In addition to higher frequencies of ST-segment elevation MI and LV dysfunction in the TVP group, there was a predominance of LAD dissection. This has been postulated to arise from the disparity in ventricular wall dynamics between the hypokinetic or dyskinetic segments affected by TVP and the remaining hyperkinetic segments, which potentially intensifies shear stress on the LAD as it traverses the anterior interventricular groove spanning from the basal to apical segments, thereby increasing its vulnerability to dissection [4], [5]. Consistent with previous research, the study also observed a greater likelihood of impaired myocardial perfusion due to abnormal microvascular circulation in TVP patients [12]. More importantly, coexistent TVP was independently linked to an adverse prognosis due to new in-hospital arrhythmias and HF events, which has been postulated to reflect substantially increased levels of endogenous stress in this subset [13]. Given the prognostic implications, clinicians caring for patients with SCAD should be vigilant for the potential concurrence of TVP.

Post-discharge medical management in SCAD is primarily guided by disease-specific considerations, particularly the risk of recurrent dissection and the presence of LV dysfunction. Following SCAD, the administration of beta-blockers is recommended to protect against recurrence by mitigating arterial shear stress via reduction of myocardial contractility and blood pressure [14]. In contrast, the role of beta-blockers in the long-term management of TVP remains uncertain due to conflicting evidence [15], [16], [17], [18], [19], [20]. For renin–angiotensin system inhibition, current SCAD guidance supports the use of ACEIs or ARBs primarily in patients with concomitant LV systolic dysfunction, consistent with general heart failure management principles [21], [22], [23]. Although the use of ACEIs or ARBs in TVP patients is supported by observational data suggesting a lower rate of mortality or recurrence [15], [16], extrapolation of these findings to SCAD should be approached cautiously given fundamental differences in underlying mechanisms. This study demonstrated that the majority of SCAD patients were prescribed beta-blockers at discharge, and patients with coexistent TVP were more commonly managed with ACEIs or ARBs compared to the non-TVP group. Nevertheless, the long-term benefits of medical therapies for patients with TVP or SCAD have yet to be validated in randomized trials.

## 5 Limitations

The study participants in the iSCAD Registry were recruited from academic institutions by cardiologists with expertise in SCAD and its associated complications. In the present study, only 39.3% of the SCAD patients underwent both coronary angiography and left ventriculography. Other imaging modalities that could potentially identify missed cases of TVP were unavailable for core lab adjudication. Consequently, the risk of referral bias and sampling bias cannot be excluded, which might have influenced the reported incidence of coexistent TVP and in-hospital events, thus limiting the generalizability of the study findings. Regarding coronary angiography, demarcating the perfusion territory of dissected coronary arteries could be challenging in instances where SCAD affects coronary branches of smaller caliber or involves tapering of terminal vessels. Regarding left ventriculography, the precision in delineating the extent of WMA in the left ventricular segments might have been compromised in instances of inadequate cine run duration or insufficient contrast injection. As previously noted in this manuscript, in the absence of follow-up imaging to confirm resolution of the wall motion abnormalities a definitive diagnosis of TTS cannot be firmly established. Additionally, identification of TVP was based upon a blinded review of coronary angiography and left ventriculography, performed without access to clinical context, cardiac biomarker results, electrocardiography findings, or other cardiac imaging modalities. These factors might have influenced the accuracy in ascertaining the presence of coexistent TVP within the SCAD cohort.

## 6 Conclusions

Among patients with SCAD in the iSCAD Registry, the presence of WMAs consistent with concomitant TVP may represent a subset exhibiting an increased risk of experiencing new arrhythmias or heart failure during hospitalization. Further investigations are warranted to gain deeper insights into the long-term prognosis and optimize clinical management strategies for this specific population.

## CRediT authorship contribution statement

**Gerald Chi:** Writing – review & editing, Writing – original draft, Project administration, Formal analysis, Data curation, Conceptualization. **Esther S.H. Kim:** Writing – review & editing, Writing – original draft, Methodology, Investigation, Formal analysis, Data curation, Conceptualization. **Heather L. Gornik:** Writing – original draft, Supervision, Investigation, Data curation. **Anna Grodzinsky:** Writing – review & editing, Writing – original draft, Validation, Methodology. **Bryan J. Wells:** Writing – original draft, Supervision, Methodology, Investigation. **Jennifer Lewey:** Writing – review & editing, Writing – original draft, Project administration, Methodology, Investigation. **Daniella Kadian-Dodov:** Writing – review & editing, Writing – original draft, Resources, Conceptualization. **Angela M. Taylor:** Writing – review & editing, Writing – original draft, Methodology, Investigation. **Connie N. Hess:** Writing – review & editing, Writing – original draft, Supervision, Methodology, Investigation. **Lori M. Tam:** Writing – review & editing, Writing – original draft, Investigation. **Rina Mauricio:** Writing – review & editing, Writing – original draft, Investigation, Conceptualization. **Stanislav Henkin:** Writing – review & editing, Writing – original draft, Methodology, Investigation. **Kathryn J. Lindley:** Writing – original draft, Supervision, Investigation, Data curation. **James L. Orford:** Writing – review & editing, Writing – original draft, Supervision, Conceptualization. **Sonia Tolani:** Writing – review & editing, Writing – original draft, Methodology, Investigation. **Fahad Alkhalfan:** Writing – review & editing, Writing – original draft, Methodology, Investigation, Conceptualization. **Cassandra Reyes:** Writing – review & editing, Writing – original draft, Project administration, Investigation. **C. Michael Gibson:** Writing – original draft, Visualization, Validation, Investigation, Conceptualization. **Katherine K. Leon:** Writing – review & editing, Writing – original draft, Methodology, Investigation, Conceptualization. **Sahar Naderi:** Writing – review & editing, Writing – original draft, Supervision, Investigation, Data curation, Conceptualization. **Nandita S. Scott:** Writing – review & editing, Project administration, Investigation. **Malissa J. Wood:** Writing – original draft, Validation, Methodology, Investigation, Data curation, Conceptualization.

## Funding

SCAD Alliance is the sole funder of this work.

## Declaration of competing interest

The authors declare the following financial interests/personal relationships which may be considered as potential competing interests:

Katherine K. Leon reports financial support was provided by SCAD Alliance. Gerald Chi reports a relationship with 10.13039/100004326Bayer Corporation that includes: funding grants. Gerald Chi reports a relationship with Janssen Science that includes: funding grants. Gerald Chi reports a relationship with 10.13039/100008322CSL Behring that includes: funding grants. Esther S.H. Kim reports a relationship with SCAD Alliance that includes: board membership. Daniella Kadian-Dodov reports a relationship with Boston Scientific Corporation that includes: consulting or advisory. Daniella Kadian-Dodov reports a relationship with Abbott Laboratories that includes: consulting or advisory. Connie N. Hess reports a relationship with CPC Clinical Research that includes: employment. C. Michael Gibson reports a relationship with SCAD Alliance that includes: non-financial support. Katherine K. Leon reports a relationship with SCAD Alliance that includes: board membership and employment. Sahar Naderi reports a relationship with SCAD Alliance that includes: board membership. Nandita S. Scott reports a relationship with SCAD Alliance that includes: board membership. Malissa J. Wood reports a relationship with SCAD Alliance that includes: board membership. If there are other authors, they declare that they have no known competing financial interests or personal relationships that could have appeared to influence the work reported in this paper.

## Appendix A Supplementary data

Supplementary Table 2 Patient characteristics, clinical presentations, imaging findings, and management for SCAD in classic TVP group versus TVP variants or non-TVP group.
