# Supplementary material for: Patient characteristics and incidence of in-hospital events in spontaneous coronary artery dissection and suspected concurrent Takotsubo syndrome: A report of the iSCAD registry
Source: Am Heart J Plus. 2026 May 9;66:100793. doi: 10.1016/j.ahjo.2026.100793 (PMC13199964; doi:10.1016/j.ahjo.2026.100793)
Supplement: Supplementary Table 2 — Patient characteristics, clinical presentations, imaging findings, and management for SCAD in classic TVP group versus TVP variants or non-TVP group. [file mmc1.docx]

**Supplementary Table 2.** Patient characteristics, clinical presentations, imaging findings, and management for SCAD in classic TVP group versus TVP variants or non-TVP group

| **Variable** | **Classic TVP (N=39)** | **TVP variants or non-TVP (N=220)** | **P-value** |
| --- | --- | --- | --- |
| **Patient characteristics** |  |  |  |
| Age at first SCAD, Mean (SD) | 52.5 (11.2) | 49.8 (10.3) | 0.207 |
| Body mass index, Mean (SD) | 27.5 (6.8) | 28.1 (9.3) | 0.642 |
| Female | 38/38 (100.0%) | 207 (94.1%) | 0.124 |
| White | 36 (92.3%) | 201 (91.4%) | 0.845 |
| Smoking | 13 (33.3%) | 55/218 (25.2%) | 0.291 |
| Diabetes | 0 (0.0%) | 3 (1.4%) | 0.463 |
| Hyperlipidemia | 6 (15.4%) | 32 (14.5%) | 0.891 |
| Hypertension | 15 (38.5%) | 52 (23.6%) | 0.051 |
| Previous myocardial infarction | 4/36 (11.1%) | 38/200 (19.0%) | 0.255 |
| Anxiety | 14 (35.9%) | 62 (28.2%) | 0.329 |
| Depression | 10 (25.6%) | 46 (20.9%) | 0.508 |
| Recreational substance use | 4 (10.3%) | 24/218 (11.0%) | 0.889 |
| Emotional stress when experiencing SCAD | 5 (12.8%) | 28 (12.7%) | 0.987 |
| Physical stress when experiencing SCAD | 6 (15.4%) | 25 (11.4%) | 0.476 |
| Extracoronary vascular abnormalities | 21/34 (61.8%) | 103/193 (53.4%) | 0.365 |
| **Clinical presentations** |  |  |  |
| ST-segment elevation MI | 21 (53.8%) | 65 (29.5%) | 0.003 |
| Non-ST-segment elevation MI | 13 (33.3%) | 116 (52.7%) | 0.026 |
| Unstable angina | 3 (7.7%) | 43 (19.5%) | 0.074 |
| Cardiogenic shock | 0 (0.0%) | 2 (0.9%) | 0.550 |
| Cardiac arrest | 5 (12.8%) | 14 (6.4%) | 0.154 |
| **Echocardiographic findings** |  |  |  |
| LVEF, Mean (SD) | 46.1 (9.3) | 55.3 (10.4) | <0.001 |
| LV dysfunction (LVEF <50%) | 23/34 (67.6%) | 34/173 (19.7%) | <0.001 |
| **Angiographic findings** |  |  |  |
| Involvement of left main artery | 1 (2.6%) | 0 (0.0%) | 0.017 |
| Involvement of left anterior descending artery | 36 (92.3%) | 124 (56.4%) | <0.001 |
| Involvement of left circumflex artery | 7 (17.9%) | 83 (37.7%) | 0.017 |
| Involvement of right coronary artery | 3 (7.7%) | 61 (27.7%) | 0.008 |
| Multivessel involvement | 5 (12.8%) | 44 (20.0%) | 0.291 |
| Type 1 SCAD | 2 (5.1%) | 27 (12.3%) | 0.192 |
| Type 2 SCAD | 29 (74.4%) | 161 (73.2%) | 0.878 |
| Type 3 SCAD | 10 (25.6%) | 53 (24.1%) | 0.835 |
| Abnormal TIMI Flow Grade | 20 (51.3%) | 100 (45.5%) | 0.501 |
| Abnormal TIMI Myocardial Perfusion Grade | 27 (69.2%) | 110 (50.0%) | 0.027 |
| **LVG findings** |  |  |  |
| WMA in anterobasal segment | 4 (10.3%) | 23/217 (10.6%) | 0.949 |
| WMA in anterolateral segment | 33 (84.6%) | 65/217 (30.0%) | <0.001 |
| WMA in apical segment | 38 (97.4%) | 83/216 (38.4%) | <0.001 |
| WMA in diaphragmatic segment | 32 (82.1%) | 59/217 (27.2%) | <0.001 |
| WMA in posterobasal segment | 2 (5.1%) | 22/217 (10.1%) | 0.323 |
| **Management** |  |  |  |
| Management for SCAD |  |  | 0.007 |
| Medical therapy only | 21/38 (55.3%) | 165/216 (76.4%) |  |
| Revascularization | 17/38 (44.7%) | 51/216 (23.6%) |  |
| Medications at discharge |  |  |  |
| ACEI/ARB | 19 (48.7%) | 60 (27.3%) | 0.007 |
| Beta blocker | 33 (84.6%) | 182 (82.7%) | 0.772 |
| Calcium channel blocker | 3 (7.7%) | 24 (10.9%) | 0.545 |
| Diuretic | 4 (10.3%) | 14 (6.4%) | 0.378 |
